# Novel alternative splicing isoform biomarkers identification from high-throughput plasma proteomics profiling of breast cancer

**DOI:** 10.1186/1752-0509-7-S5-S8

**Published:** 2013-12-09

**Authors:** Fan Zhang, Mu Wang, Tran Michael, Renee Drabier

**Affiliations:** 1 Department of Academic and Institutional Resources and Technology, University of North Texas Health Science Center, Fort Worth, USA; 2 Department of Forensic and Investigative Genetics, University of North Texas Health Science Center, Fort Worth, USA; 3 Department of Biochemistry and Molecular Biology, IU School of Medicine, Indianapolis, IN 46202, USA; 4 Indiana Center for Systems Biology and Personalized Medicine, Indianapolis, IN 46202, USA

## Abstract

**Background:**

In the biopharmaceutical industry, biomarkers define molecular taxonomies of patients and diseases and serve as surrogate endpoints in early-phase drug trials. Molecular biomarkers can be much more sensitive than traditional lab tests. Discriminating disease biomarkers by traditional method such as DNA microarray has proved challenging. Alternative splicing isoform represents a new class of diagnostic biomarkers. Recent scientific evidence is demonstrating that the differentiation and quantification of individual alternative splicing isoforms could improve insights into disease diagnosis and management. Identifying and characterizing alternative splicing isoforms are essential to the study of molecular mechanisms and early detection of complex diseases such as breast cancer. However, there are limitations with traditional methods used for alternative splicing isoform determination such as transcriptome-level, low level of coverage and poor focus on alternative splicing.

**Results:**

Therefore, we presented a peptidomics approach to searching novel alternative splicing isoforms in clinical proteomics. Our results showed that the approach has significant potential in enabling discovery of new types of high-quality alternative splicing isoform biomarkers.

**Conclusions:**

We developed a peptidomics approach for the proteomics community to analyze, identify, and characterize alternative splicing isoforms from MS-based proteomics experiments with more coverage and exclusive focus on alternative splicing. The approach can help generate novel hypotheses on molecular risk factors and molecular mechanisms of cancer in early stage, leading to identification of potentially highly specific alternative splicing isoform biomarkers for early detection of cancer.

## Introduction

A biomarker as defined by the National Cancer Institute is "a biological molecule found in blood, other body fluids, or tissues that is a sign of a normal or abnormal process, or of a condition or disease [1]." It is a characteristic that is objectively measured and evaluated as an indicator of normal biological processes, pathogenic processes, or pharmacologic responses to a therapeutic intervention [2]. The field of biomarkers has grown extensively over the past decade in many areas such as medicine, cell biology, genetics, geology and astrobiology, and ecotoxicology etc. and biomarkers are currently being studied in many academic centers and in industry.

In recent years, functional genomics studies using DNA Microarrays have been shown effective in identifying markers differentiating between breast cancer tissues and normal tissues, by measuring thousands of differentially expressed genes simultaneously [3-5].

However, early detection and treatment of breast cancer is still challenging. One reason is that obtaining tissue samples for microarray analysis can be still difficult. Another reason is that genes are not directly involved in any physical functions. On the contrary, the proteome, are the real functional molecules and the keys to understanding the development of cancer. Moreover, the fact that breast cancer is not a single homogeneous disease but consists of multiple disease status, each arising from a distinct molecular mechanism and having a distinct clinical progression path [6] makes the disease difficult to early detect.

Alternative splicing isoforms represent a new class of diagnostic biomarkers [7]. The chance of success with alternative splicing isoforms would be higher than the conventional approach [8,9]. Alternative splicing occurs in 95% human genes and works by selecting specific exons and sometimes even intronic regions of the gene into mature mRNAs [10]. Alternative splicing accounts for approximately 8% of all protein isoforms which is any of several different forms of the same protein and have three types: alternative splicing, SNP, and posttranslational modification (PTM).

Recent scientific studies have shown that diseased cells may produce many types of splicing variants of common regulatory proteins, e.g., protein kinase C, 14-3-3, p53, and VGFR, which could provide novel insights into complex disease diagnosis and management, particularly in cancers [11-14]. Alternative mRNA splicing is an important source of achieving molecular functional diversity. It is often regulated in a temporal or tissue-specific fashion, giving rise to different protein isoforms in different tissues or developmental states mediated by extracellular signaling mechanisms [15,16]. Splicing regulation is a key mechanism to tune gene expression to a variety of conditions and its dysfunction may often be at the basis of the onset of genetic disease and cancer [9]. In cancer, many examples of alternative splicing isoforms were reported [17-21]. For example, Julian et al. used a high-throughput reverse transcription-PCR-based system for splicing annotation to monitor the alternative splicing profiles of 600 cancer-associated genes in a panel of 21 normal and 26 cancerous breast tissues. They found that 41 alternative splicing events significantly differed in breast tumors relative to normal breast tissues and that most cancer-specific changes in splicing that disrupt known protein domains support an increase in cell proliferation or survival consistent with a functional role for alternative splicing in cancer. Compared to normal mRNA splicing events, alternative splicing mechanisms and patterns in complex diseases such as cancer can be quite complex. Finding alternative splicing isoforms or patterns of their development, therefore, have been promising in helping develop high-quality biomarkers and targets for disease management [22].

Discovering disease biomarkers in the human plasma has been met with both enthusiasm and criticisms in recent years. On one hand, it is expected that disease conditions such as cancer may be diagnosed early by analyzing complex protein mixtures in easily accessible human blood (serum or plasma), in which proteins induced by cancer may be differentially cleaved, secreted, leaked out, and therefore differentially detected from normal healthy conditions. On the other hand, the bulk of proteins circulating in human blood vary in different conditions without cancers and across different individuals, and changes in protein expressions are often diluted in the blood -- extremely challenging for biological interpretation of protein quantification changes [23,24]. The use of alternative splicing isoforms as potential biomarkers, therefore, offers a new opportunity to use the detectability instead of quantification of biomarker peptides, for the peptides that may map to unique alternative splicing isoforms specific to cancer.

However, systematic, comprehensive, proteome-scale experimental and computational characterization of protein isoforms directly at the protein/peptide level and with exclusive focus on alternative splicing has never been reported. Compared with the "indirect" transcriptome-level characterizations such as EST sequencing[25], exon array[26], exon-exon junction array[27], and next-generation sequencing of all mRNA transcripts [28,29], "direct" proteome-level characterization of alternative splicing isoforms can address the wide-spread concern that mRNA expressions and protein expression do not correlate well in a clinical proteomics [30,31]. In 2010, we developed the PEPtidomics Protein Isoform Database (PEPPI [32], http://bio.informatics.iupui.edu/peppi), a database of computationally-synthesized human peptides that can identify protein isoforms derived from either alternatively spliced mRNA transcripts or SNP variations. Although the PEPPI database is the first peptidomics protein isoform database, it is not exclusive to alternative splicing and has poor coverage of alternative splicing.

Therefore, based on the PEPPI [32], we presented a new peptidomics approach to searching novel alternative splicing isoform in proteomics data and demonstrated that the approach can help researchers identify and characterize novel alternative splicing isoform from experimental proteomics data and discover diagnostic value and clinical significance.

## Materials and methods

### Human plasma samples

Plasma protein profiles were collected by the Hoosier Oncology Group (HOG) (Indianapolis, IN, USA). Each sample was analyzed in a single batch by mass spectrometry. In the study, 80 plasma samples were collected (40 samples collected from women with breast cancer and 40 from healthy volunteer woman who served as controls). An independent validation dataset of 80 samples which contains 40 samples collected from women diagnosed with breast cancer and 40 from healthy volunteer woman who served as controls were collected by the Hoosier Oncology Group (HOG) (Indianapolis, IN, USA) too and is comparable to the study in the demography and clinical distribution of breast cancer stages/subtypes. For example, most of patients involved in the two studies were diagnosed with an early stage breast cancer (stage I or II), fell into age group between 40 and 65, and had mean tumor size of 2.2.

### Protein identification and quantification

For protein identification, Tryptic peptides were analyzed using Thermo-Fisher Scientific linear ion-trap mass spectrometer (LTQ) coupled with a Surveyor HPLC system. Peptides were eluted with a gradient from 5 to 45% Acetonitrile developed over 120 minutes and data were collected in the *triple-play *mode (MS Scan, zoom scan, and MS/MS scan). The acquired raw peak list data were generated by XCalibur (version 2.0) using default parameters and further analyzed by the label-free identification and quantitative algorithm using default parameters described by Higgs *et al *[33]. MS database searches were performed against the combined protein data set from International Protein Index and the non-redundant NCBI-nr human protein database, which totaled 22,180 protein records. Carious data processing filters for protein identification were applied to keep only peptides with the XCorr score above 1.5 for singly charged peptides, 2.5 for doubly charged peptides, and 3.5 for triply charged peptides. These XCorr scores were set according to linear discriminant analysis similar to that described in DTASelect (version 2.0) to control false-positive rate at below 5% levels.

For protein quantification, first, all extracted ion chromatograms (XICs) were aligned by retention time. Each aligned peak were matched by precursor ion, charge state, fragment ions from MS/MS data, and retention time within a one-minute window. Then, after alignment, the area-under-the-curve (AUC) for each individually aligned peak from each sample was measured, normalized, and compared for relative abundance--all as described in [33]. Here, a linear mixed model generalized from individual ANOVA (Analysis of Variance) was used to quantify protein intensities. In principle, the linear mixed model considers three types of effects when deriving protein intensities based on weighted average of quantile-normalized peptide intensities: 1) *group effect*, which refers to the fixed non-random effects caused by the experimental conditions or treatments that are being compared; 2) *sample effect*, which refers to the random effects (including those arising from sample preparations) from individual biological samples within a group; 3) *replicate effect*, which refers to the random effects from replicate injections from the same sample preparation.

### Peptidomics approach to searching novel alternative splicing isoform in proteomics data

**Figure 1:**
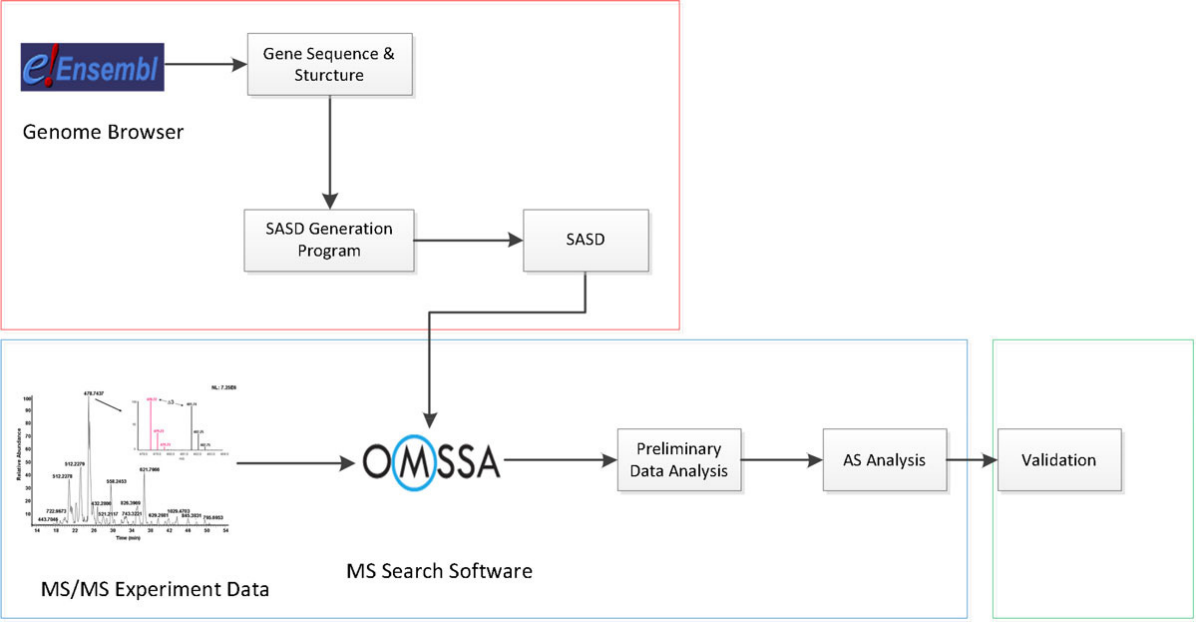
**A peptidomics approach to identifying novel alternative splicing isoforms in proteomics data**.

Our peptidomics approach to identifying novel alternative splicing isoform in proteomics data includes three steps (Figure 1): 1) building an synthetic database of alternative splicing isoforms for proteomics experiments; 2) identification, characterization of alternative splicing isoform using proteomics; and 3) validation of alternative splicing isoform.

### Step 1: building a synthetic database of alternative splicing isoforms for proteomics experiments

Briefly, we developed the **S**ynthetic **A**lternative **S**plicing **D**atabase (**SASD**) [34] in three steps: 1)extracting information on gene structures of all genes in the human genome and incorporating the IPAD database [35], 2)compiling artificial splicing transcripts, and 3) translating the artificial transcripts into alternative splicing peptides.

In the first step, we use the BioMart to extract information on all human genes in the Ensemble [36] from the Homo sapiens genes dataset (GRCh37.p10) in the Ensembl Genes 71 database. We then extract information on each human gene's position, name, exon/intron coordinates, exon phase, sequences, and annotation.

In the second step, we generate artificial splicing transcript (AST), which is an exhaustive compilation of two categories of peptides. The first category is the peptides translated from all single exons and introns, and the second category is the peptides that cover all theoretically possible exon/intron junction regions of all genes in the human genome. The first category contains two types of alternative splicing: single exon(EXON_NM) and single intron (INTRON_AS). The second category contains four types of alternative splicing: intron-exon (I_E_AS, left intron retention junction), exon-intron (E_I_AS, right intron retention junction), neighboring exon-exon (E_E_NM, normal splicing junction) and non-neighboring exon-exon (E_E_AS, exon skipping junction).

In the third step, we directly use the phase to translate the sequence for the exons with the phase information in Ensemble transcript. For the exons without the phase information in Ensemble transcript, three open reading frames are used in translating into three peptides and the longest peptide crossing the splicing site is reserved as alternative splicing peptide for SASD.

### Step 2: identification and characterization of alternative splicing isoform using proteomics

The most popular three types of search algorithms are 1) correlating acquired MS/MS with theoretical spectrum, counts the number of peaks in common, such as: SEQUEST [37] and X!Tandem [38], 2) modeling the extent of peptide fragmentation, then estimates the probability that an assignment is incorrect due specifically to a random match, such as Mascot [39] and OMSSA [40], and 3) De Novo Sequencing such as Lutefisk[41] and PEAKS[42]. We first run OMSSA against SASD to identify peptide from MS data. Then we perform preliminary data analysis. Last, we extract information about alternative splicing.

OMSSA reports hits ranked by E-value. An E-value for a hit is a score that is the expected number of random hits from a search library to a given spectrum, such that the random hits have an equal or better score than the hit. For example, a hit with an E-value of 1.0 implies that one hit with a score equal to or better than the hit being scored would be expected at random from a sequence library search [40]. The E-value is calculated to report the expected frequency of observing scores equivalent to or better than the one for the reported peptide if the results were to take place randomly. The lower the E-value is, the more significant the score for the identified peptide by the peptide search using SASD database is.

One-sided Wilcoxon signed-rank test is used to perform the preliminary statistical analysis in order to identify peptides with significant occurrence differences in the health and breast cancer samples.

### Step 3: validation of alternative splicing isoform

We present two kinds of methods to validate isoforms in proteomics data, which are 1) literature curation of alternative splicing isoforms and 2) cross-validation of multiple studies. First, we perform an extensive literature curation to determine the constituents of alternative splicing isoform. Then, we validate results using independent proteomics datasets derived from other study. We believe that such an integrative systems approach is essential to development and validation of panel alternative splicing isoform that may withstand rigorous testing for the future steps.

#### Pathway analysis

Pathway analysis is performed using the following databases: Integrated Pathway Analysis Database (IPAD) (http://bioinfo.hsc.unt.edu/ipad/) [43].

## Results

The 80 breast cancer plasma samples with 40 samples from women diagnosed with breast cancer and 40 from healthy volunteer women as controls were searched by OMSSA[40] against the SASD database. After OMSSA searching, preliminary statistical analysis, and alternative splicing, we identified the eight alternative splicing isoform biomarkers using the peptidomics approach to searching novel alternative splicing isoform in the proteomics data (Table 1). The peptides with E-value greater than 0.01 were filtered out. The P-value was calculated by performing one-sided Wilcoxon signed-rank test to examine the probability that the median difference between two groups of samples is greater than zero. The number of such peptide found in Health (h) and Cancer (c) samples are listed separately in the table. The tested peptides are more likely to exist in cancer samples than in healthy samples when P-value is small. Bold text is the left part of the junction and italic text is the right part. Splicing site is marked by ^ or (). '()' means the splicing site is shared by the left region and right region. For example, the second peptide **QTPKHISESLGAEVDPDMSWSSSLATPPTLSSTVLI**(G)*LLHSSVK *is a synthetic product of the ENST00000380152 in gene BRCA2 when its eighth, ninth, and tenth exons are skipped and its seventh exon is combined together with its eleventh exon. The Glycine is the shared splicing site between the seventh exon and the eleventh exon.

**Table 1:** Novel alternative splicing isoform candidate biomarkers for breast cancer in plasma

Peptide sequence	gene	transcript	mode	type	pvalue	h	c	Peptide
								Atlas
SWGGRPQRMGAVPGGVWSAVLMGGAR	ERBB2	ENST00000269571	i18	INTRON_AS	9.48E-05	4	20	No
**QTPKHISESLGAEVDPDMSWSSSLATPPTLSSTVLI**(G)*LLHSSVK*	BRCA2	ENST00000380152	E7_E11	E_E_AS	8.57E-04	1	12	No
**SLWLQSQPHFCCFWLTVTFPPPLQ**^*THRELAQSSHAQR*	NTRK3	ENST00000317501	i2_E3	i_E_AS	1.22E-02	2	10	No
**WGLLLALLPPGAASTQ**(A)*VWTWMTR*	ERBB2	ENST00000269571	E1_E16	E_E_AS	1.22E-02	2	10	No
LSWNHVARALTLTQSLVSSVTSGK	NTRK3	ENST00000559764	i2	INTRON_AS	1.39E-02	4	13	No
**CQ**(G)*EPYHDIRFNLMAVVPDR*	BAP1	ENST00000460680	E3_E9	E_E_AS	3.33E-02	9	18	No
**QVLP**^*VGVLGPPGQQAPPPYPGPHPAGPPVIQQPTTPMFVAPPPK*	PBRM1	ENST00000296302	E9_E29	E_E_AS	3.89E-02	6	14	No
**DHLACW**^*DYDLCITCYNTKNHDHK*	EP300	ENST00000263253	E22_E31	E_E_AS	4.50E-02	4	11	No

None of the eight peptides are reported to have ever been detected in the Peptide Atlas database, which contains a comprehensive catalogue of all peptides derived from published proteomics experiments. They are not found with normal splicing mechanism (Table 1).

**Figure 2:**
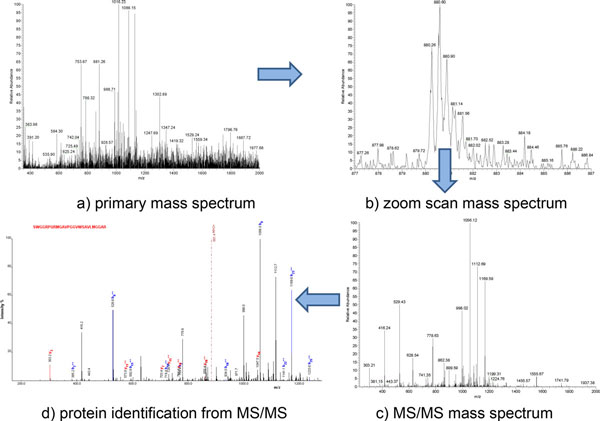
**Triple play mode spectrum of SWGGRPQRMGAVPGGVWSAVLMGGAR in patient C_024**.

The first sequence is a single intron alternative splicing. It was observed in 20 patient samples and 4 healthy samples. Triple play mode of the annotated Thermo-Finnegan LCQ-DECA ion-trap MS/MS spectrum is shown in Figure 2[44]. The triple play mode includes a) primary mass spectrum; b) zoom scan mass spectrum; c) MS/MS mass spectrum and d) protein identification from MS/MS). Due to space limit, the spectrums of other seven alternative splicing sequences are omitted.

**Figure 3:**
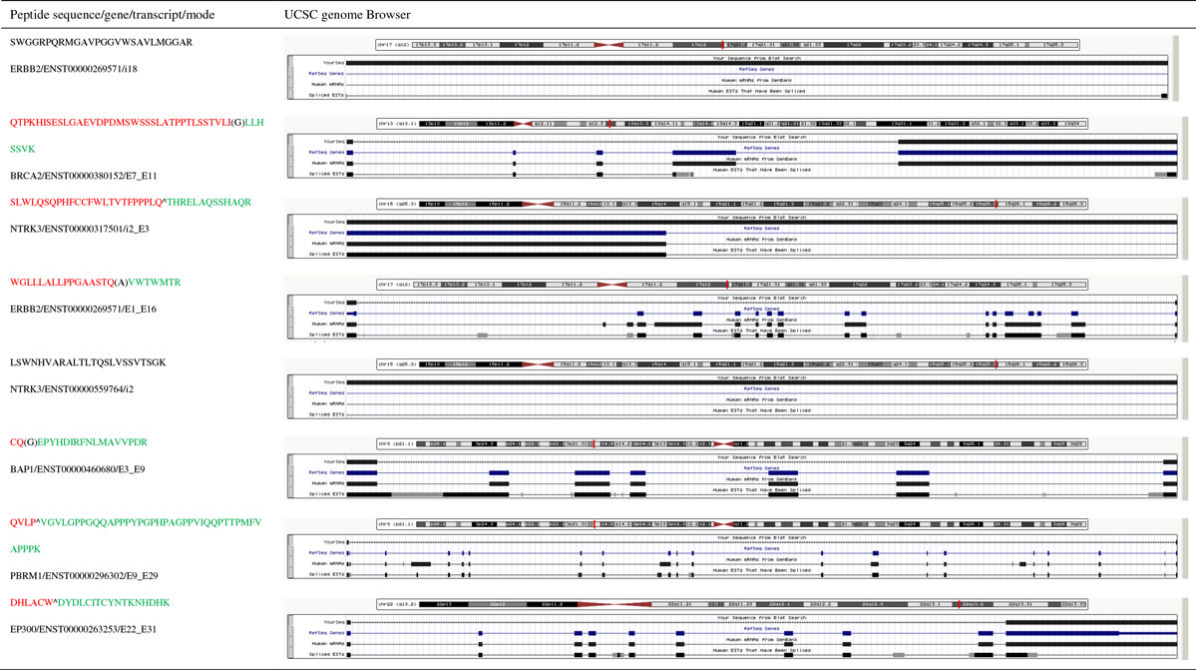
**UCSC genome browser screen shot of genomic region for the novel peptide**.

The eight peptides, identified by OMSSA, have significant difference in the numbers of hit samples between healthy women and breast cancers (pvalue < 0.05, Table 1). A screen shot from the UCSC genome browser [45] in the region of these peptides are also shown in Figure 3. It shows that these peptide sequences are not found in EST sequences and mRNA from Genbank and one refseq gene(Figure 3).

Table 1 shows that our peptidomics approach has significant potential in enabling discovery of new types of high-quality alternative splicing isoform biomarkers. Further literature search found that there are no any literature reports for the eight peptides. Moreover, a cross-validation found that identification of the eight peptides is supported by the independent Study which contains 40 samples collected from women diagnosed with breast cancer and 40 from healthy volunteer woman who served as controls.

Pathway analysis shows the pathways linked with the eight alternative splicing isoforms are transcription factor, signaling, cancer, and synthesis (Table 2).

**Table 2:** Pathway analysis for the eight alternative splicing isoforms.

PathwayID	PathwayName	Molecule
200017	p73 transcription factor network	BRCA2;EP300
200141	FOXM1 transcription factor network	EP300;BRCA2
200200	Validated targets of C-MYC transcriptional repression	EP300;ERBB2
hsa04520	Adherens junction	ERBB2;EP300
hsa05212	Pancreatic cancer	ERBB2;BRCA2
1306955	GRB7 events in ERBB2 signaling	ERBB2
1358803	Downregulation of ERRB2:ERBB3 signaling	ERBB2
168253	Host Interactions with Influenza Factors	PBRM1
168268	Virus Assembly and Release	PBRM1
168270	Fusion and Uncoating of the Influenza Virion	PBRM1
168275	Entry of Influenza Virion into Host Cell via Endocytosis	PBRM1
168303	Packaging of Eight RNA Segments	PBRM1
168330	Viral RNP Complexes in the Host Cell Nucleus	PBRM1
192814	vRNA Synthesis	PBRM1
192869	cRNA Synthesis	PBRM1
200159	ErbB receptor signaling network	ERBB2
168277	Influenza Virus Induced Apoptosis	PBRM1
168288	Fusion of the Influenza Virion to the Host Cell Endosome	PBRM1
168298	Release	PBRM1
168302	Budding	PBRM1
168336	Uncoating of the Influenza Virion	PBRM1
192905	vRNP Assembly	PBRM1
73951	Homologous recombination repair of replication-independent double-strand breaks	BRCA2
918233	TRAF3-dependent IRF activation pathway	EP300
73888	Homologous Recombination Repair	BRCA2
76003	Presynaptic phase of homologous DNA pairing and strand exchange	BRCA2
76010	Homologous DNA pairing and strand exchange	BRCA2
h_hifPathway	Hypoxia-Inducible Factor in the Cardiovascular System	EP300
h_pitx2Pathway	Multi-step Regulation of Transcription by Pitx2	EP300
h_ppargPathway	Role of PPAR-gamma Coactivators in Obesity and Thermogenesis	EP300
h_RELAPathway	Acetylation and Deacetylation of RelA in The Nucleus	EP300
hsa05200	Pathways in cancer	ERBB2;BRCA2;EP300
h_carm1Pathway	Transcription Regulation by Methyltransferase of CARM1	EP300
h_melanocytepathway	Melanocyte Development and Pigmentation Pathway	EP300
h_pelp1Pathway	Pelp1 Modulation of Estrogen Receptor Activity	EP300
h_vdrPathway	Control of Gene Expression by Vitamin D Receptor	EP300
hsa05215	Prostate cancer	ERBB2;EP300
h_il7Pathway	IL-7 Signal Transduction	EP300
168325	Viral Messenger RNA Synthesis	PBRM1
1963640	GRB2 events in ERBB2 signaling	ERBB2
419524	Fanconi Anemia pathway	BRCA2
73890	Double-Strand Break Repair	BRCA2
416572	Sema4D induced cell migration and growth-cone collapse	ERBB2
h_g2Pathway	Cell Cycle: G2/M Checkpoint	EP300
h_mef2dPathway	Role of MEF2D in T-cell Apoptosis	EP300
h_atrbrcaPathway	Role of BRCA1, BRCA2 and ATR in Cancer Susceptibility	BRCA2
h_her2Pathway	Role of ERBB2 in Signal Transduction and Oncology	EP300
h_nthiPathway	NFkB activation by Nontypeable Hemophilus influenzae	EP300
h_p53hypoxiaPathway	Hypoxia and p53 in the Cardiovascular system	EP300
h_tgfbPathway	TGF beta signaling pathway	EP300
1250196	SHC1 events in ERBB2 signaling	ERBB2
200025	Signaling events mediated by HDAC Class III	EP300
hsa03440	Homologous recombination	BRCA2
200164	Retinoic acid receptors-mediated signaling	EP300
400685	Sema4D in semaphorin signaling	ERBB2
933541	TRAF6 mediated IRF7 activation	EP300

## Discussions

We described the peptidomics approach to searching novel alternative splicing isoform in proteomics data, especially artificial alternative splicing and SNP. We can use it to identify two types of common alternative splicing events: Exon Skipping and Intron Retention. Exon Skipping is an alternative splicing mechanism in which exon(s) are included or excluded from the final gene transcript leading to extended or shortened mRNA variants. And Intron Retention is an event in which an intron is retained in the final transcript. Other types of alternative splicing events such as alternative 3' splice site and 5' splice site are not included in our method but can be derived indirectly from the two basic types: exon skipping and intron retention.

The current protein sequence databases used by tandem mass spectra search engines, for example IPI, UniProt, and NCBI nr, are designed to be useful as possible to as many researchers as possible. As such, they are a less than ideal substrate for tandem mass spectra search. Protein sequence databases typically represent only "full-length" protein sequences and attempt to collapse protein variants to a single "consensus" entry. Tandem mass spectra search engines, however, chop up the protein sequence using an in-silico enzymatic digestion (such as trypsin), so full-length proteins are not needed in order to identify experimentally observed peptides; and the currently available search engines require the experimental peptides' sequences be explicitly present in the sequence database in order to identify them, so explicit sequence variants are very important. The SASD is in fact a complete peptide sequence database, which includes majority of all occurrence of alternative splicing. It also provides alternative splicing for each peptide, such as splicing mode, splicing type, splicing site, starting position, ending position, and peptide sequence.

Moreover, the current protein sequence databases and some alternative splicing database such as ASTD and EID are not ideal or enough for identifying alternative splicing isoform from tandem mass spectrometry. There are either no or very few isoform information in these databases. For example, ASTD only includes 9757 occurrences of intron isoforms and 5214 occurrences of exon isoforms. Even for Cassette exons event, the number of occurrences is only 12470[15]. In contrast, the SASD database in our method includes 11,919,779 Alternative Splicing peptides covering about 56,630 genes (ensembl gene IDs), 95,260 transcripts (ensembl transcript IDs), 1956 pathways, 6704 diseases, 5615 drugs, and 52 organs.

Its comprehensive coverage means better sensitivity in identifying novel alternative splicing isoforms than the PEPPI. And its exclusive focus on alternative splicing can definitely increase the specificity of the identification of alternative splicing.

Alternative splicing isoform biomarkers are apparently important and can serve as an alternative to traditional biomarkers. We can use quantitative information such as p-value to determine the significance of the marker. We can also use the qualitative information such as: splicing type, splicing mode, peptide sequence etc. to further analyze the alternative splicing's mechanism. We think that combination of traditional biomarkers with the alternative splicing isoform biomarkers will definitely help us better understand the treatment, diagnosis, and prognosis of cancer.

## Competing interests statement

The authors declare that they have no competing interests.

## Authors' contributions

RD and FZ conceived the initial work and designed the method. FZ and RD developed the alternative splicing database and method, and performed the computational analyses. MW provided experimental data. TM performed literature search. All authors are involved in the drafting and revisions of the manuscript.

## Declarations

The publication costs for this article were funded by the bioinformatics program in University of North Texas Health Science Center.

This article has been published as part of *BMC Systems Biology *Volume 7 Supplement 5, 2013: Selected articles from the International Conference on Intelligent Biology and Medicine (ICIBM 2013): Systems Biology. The full contents of the supplement are available online at http://www.biomedcentral.com/bmcsystbiol/supplements/7/S5.
